# Gabapentin as an Adjunctive Therapy in Managing Visceral Hyperalgesia, Severe Feeding Intolerance, and Malnutrition in an Infant: A Case Presentation and Literature Review

**DOI:** 10.7759/cureus.75880

**Published:** 2024-12-17

**Authors:** Pedro Escalona, Anayah Sarkar

**Affiliations:** 1 Pediatrics, University of Florida, Pensacola, USA

**Keywords:** : delayed gastric emptying, failure-to-thrive, feeding difficulty, gabapentin neuro, gt feeds, : infant and young child feeding, visceral hyperalgesia

## Abstract

A two-month-old developmentally normal full-term female presented with severe feeding intolerance, progressive weight loss, and persistent fussiness, leading to multiple emergency department visits and eventual hospitalization. Initial evaluations, including laboratory tests and imaging, were unremarkable, prompting a series of diagnostic and therapeutic interventions. A multidisciplinary approach, including empiric gastroesophageal reflux disease (GERD) therapy, was started. A gastric emptying study, upper GI, and endoscopy were done. She underwent gastrostomy tube (G-tube) placement, during which a gastric wall perforation was identified and managed intraoperatively. Multiple attempts at nutritional management, including nasoduodenal tube placement and total parenteral nutrition, were met with limited success. Gastroparesis and visceral hyperalgesia were suspected as underlying causes.

Unlike previous cases documented in patients with a history of cardiac surgery, this neurologically normal patient had no such surgical history. A trial of gabapentin for possible visceral hyperalgesia resulted in a gradual improvement in oral tolerance and significant improvement with fussiness and discomfort. The patient was discharged at four months of age with gastrojejunal tube feeds and ongoing medical therapy. In this case, we highlight the utility of a gabapentin trial in an otherwise healthy patient. This case underscores the complexities of managing severe feeding disorders in infants, particularly the role of gabapentin in addressing suspected visceral hyperalgesia and failure to thrive in a patient without previous neurological or cardiac surgery.

## Introduction

Severe feeding intolerance in infants presents significant diagnostic and therapeutic challenges, especially when conditions like gastroparesis and visceral hyperalgesia are suspected. Failure to thrive (FTT) cases often require extensive evaluation to rule out underlying metabolic, anatomical, and neurological causes, but if, in addition, visceral hyperalgesia is suspected, it can be more complicated to manage. The management of these conditions is further complicated by the need for individualized care plans, often involving surgical, nutritional, and pharmacological interventions. Recent studies have highlighted the potential of gabapentin in improving feeding outcomes in infants with visceral hyperalgesia, FTT, and complex medical histories with minimal side effects [1]. However, unlike the currently available literature, this report details the case of a two-month-old female with severe feeding intolerance, suspected gastroparesis, visceral hyperalgesia, and malnutrition without previous neurological conditions or cardiac surgery and the role of gabapentin in addressing her FTT.

## Case presentation

**Figure 1:**
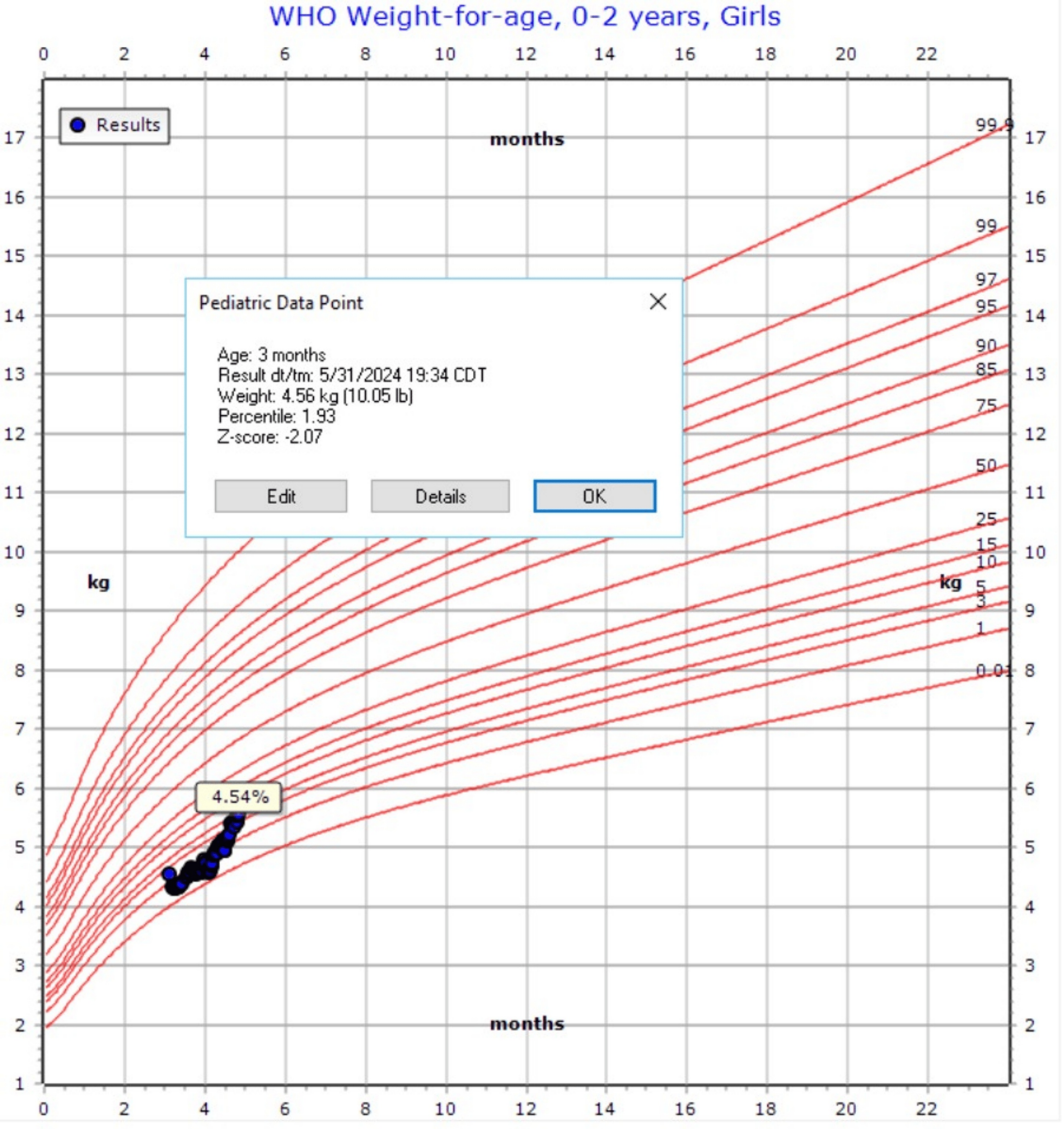
Growth chart on admission

EY is a two-month-old female, born at 38 weeks + three days via vaginal delivery, with a body weight (BW) of 2.95 kg (26th percentile) and a height in the 32nd percentile. Her birth was unremarkable, with no NICU stay, and her development is appropriate for her age. The patient was repeatedly seen in the emergency department for refusal to eat, non-bilious emesis, progressive weight loss, and intermittent fussiness. Upon admission to the inpatient pediatric service, severe protein-calorie malnutrition was diagnosed, with a weight of 4.28 kg (1st percentile) and a z-score of -2.0 (Figure 1). Initial laboratory workup and imaging studies, including assessments for hypertrophic pyloric stenosis, gastroesophageal reflux, and intra-abdominal masses, were unremarkable. A swallow study demonstrated intermittent weak sucking ability, complicating the diagnosis. Speech therapy was consulted. 

Nutritional management proved challenging, starting with breastfeeding and followed by multiple hydrolyzed infant formulas, which were tried without success for two months. Nasogastric tube (NGT) showed some tolerance with intermittent episodes of crying and vomiting despite a trial of promotility agents, reflux medications, and anti-gas medication. The patient's condition necessitated the placement of a gastrostomy tube (GT). During the procedure, the needle was observed to be at risk of perforating the anterior gastric wall. As a precautionary measure, the site was sutured to prevent a full-thickness injury. The procedure was subsequently completed without further incident. 

Postoperatively, the patient exhibited poor tolerance to GT feeds, with ongoing vomiting and feeding intolerance. Suspecting gastroparesis, erythromycin was trialed but yielded no improvement, leading to the placement of a nasoduodenal (ND) tube. Despite this intervention, feeding tolerance remained minimal. Neurological evaluations, including brain MRI and head CT, were conducted to rule out underlying central nervous system conditions but returned with normal results. 

New onset hematemesis prompted further investigation, revealing a small, clean-based ulcer between the duodenal bulb and the second portion of the duodenum. This finding led to the re-initiation of proton pump inhibitor therapy with lansoprazole in combination with famotidine. Despite repositioning the ND tube distal to the ulcer, feeding intolerance persisted, necessitating the initiation of total parenteral nutrition through a peripherally inserted central catheter (PICC) line. Total parenteral nutrition (TPN) was facilitated over six weeks and provided adequate weight gain, and a follow-up endoscopy confirmed ulcer healing.

**Figure 2:**
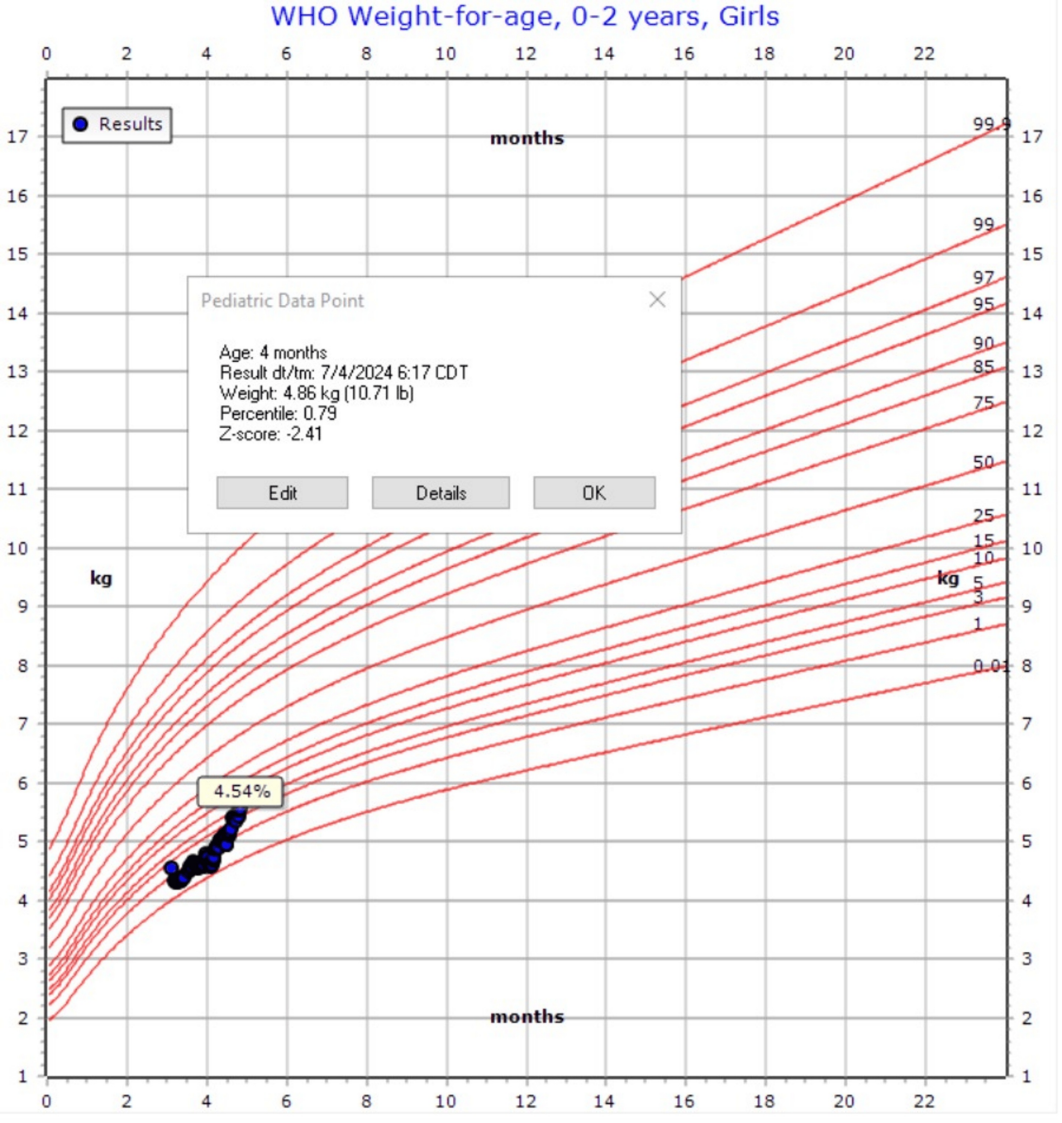
Weight at the start of gabapentin trial

**Figure 3:**
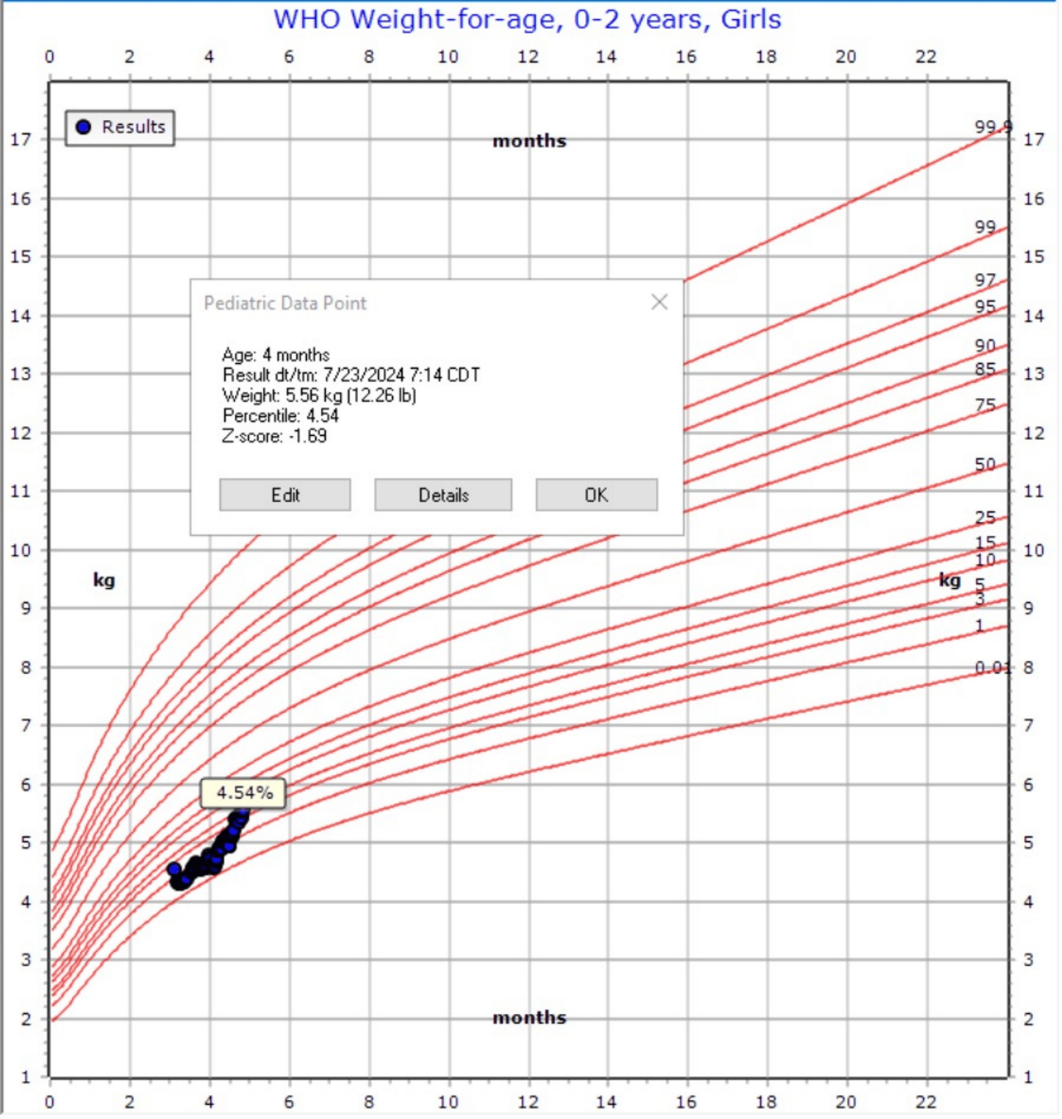
Weight at discharge on gabapentin

Given her fussiness out of proportion, vomiting, and ulcer, a trial of gabapentin was initiated at a dose taken (5 mg/kg) three times daily to address suspected visceral hyperalgesia. This resulted in a moderate improvement in gastric tolerance. Upon discharge, the GT was converted to a gastrojejunostomy (GJ) tube, leading to consistent slow weight gain with the improvement of the z-score from -2.4 to -1.69 (Figures 2-3). Gradual increases in gastric:jejunal feeds ratio were noted, and the patient would continue to advance this as an outpatient with GI, nutrition, and ST. This improvement allowed for the discontinuation of TPN and the PICC line, and the patient was eventually discharged at four months of age with GJ-tube feeds, gabapentin, and lansoprazole.

## Discussion

This case underscores the complexity of managing severe feeding intolerance in infants, particularly with concomitant conditions like visceral hyperalgesia and gastroparesis. Despite multiple interventions, including ND tube placement and TPN, the patient experienced persistent feeding intolerance. The introduction of gabapentin, informed by emerging evidence and its application in managing visceral hyperalgesia, was crucial in improving the patient’s oral tolerance. This reinforces its potential as a therapeutic option in similar cases. 

The use of gabapentin in this case was informed by emerging evidence supporting its efficacy in managing visceral hyperalgesia in neonates [2]. Visceral hyperalgesia has been described as increased pain from GI stimulus. The origin is suspected to be neuropathic, and hence why gabapentin was studied as a possible resource in management [3,4]. Recent studies have highlighted the potential of gabapentin in improving feeding outcomes in infants with complex medical histories. A large retrospective study evaluated gabapentin use in infants younger than one year, demonstrating significant improvement in weight-for-age z-scores and reduction in pain scores with minimal adverse events [1]. This study suggests that gabapentin is well-tolerated and effective in this population, supporting its use in cases of suspected visceral hyperalgesia. 

Another study focused on infants with congenital heart disease and feeding difficulties post-surgery. The study suggested that gabapentin may reduce discomfort associated with oral feeding, potentially increasing voluntary oral intake by alleviating oropharyngeal, esophageal, or gastric discomfort. Despite the limitations of the study, including its retrospective design and small sample size, the findings align with the favorable response observed in our patient case [3]. 

Another case study emphasized the importance of considering gabapentin for neonates with neurological impairments who present with symptoms of visceral hyperalgesia. The report highlighted the challenges of diagnosing and treating visceral hyperalgesia in this population and suggested gabapentin as a viable treatment option, with evidence of its safety and efficacy across multiple cases [4]. 

Lastly, a study by O'Mara et al. explored gabapentin use in neurologically intact infants with abdominal disorders, emphasizing its potential to improve feeding tolerance in this population. The study discussed the challenge of diagnosing visceral hyperalgesia, often a diagnosis of exclusion after other causes of pain or discomfort have been ruled out. Their findings suggest that gabapentin, initiated at 5 to 10 mg/kg every eight to 12 hours, may improve feeding tolerance and alleviate symptoms such as feeding intolerance, abdominal discomfort, and retching. The study highlights the importance of considering gabapentin as a treatment option for infants with abdominal disorders who exhibit signs of visceral hyperalgesia, even in the absence of neurological impairment [5]. 

The introduction of gabapentin in the management of this patient, based on published research, contributed to the observed improvement in oral tolerance in our patient. This case also emphasizes the importance of a multidisciplinary approach, combining surgical, nutritional, and pharmacological strategies to achieve a favorable outcome. Future research may explore the role of gabapentin and other neuropathic pain medications in similar cases, potentially expanding therapeutic options for infants with severe feeding disorders.

## Conclusions

This case underscores the significant challenges in diagnosing and managing severe feeding intolerance and suspected visceral hyperalgesia with gastroparesis in infants. The patient's feeding intolerance persisted despite multiple interventions, including GT placement, ND tube feeding, and total parenteral nutrition. The introduction of gabapentin, based on emerging evidence, played a crucial role in improving oral tolerance and addressing suspected visceral hyperalgesia. The patient's gradual recovery and eventual discharge with GJ-tube feeds highlight the utility of gabapentin in managing complex feeding disorders in neurologically intact patients. Future studies are needed to explore further the role of gabapentin and other neuropathic pain medications in this population, potentially expanding therapeutic options for infants with severe feeding disorders. A potential area of further study is the length of treatment as well as the optimal method for weaning/discontinuation of the medication.
